# A Cross-Sectional Study of Rehabilitation Training Intensity and Physical Restraints in Patients With Neurocognitive Disorders in an Acute Care Hospital

**DOI:** 10.7759/cureus.81027

**Published:** 2025-03-23

**Authors:** Yukako Ishida, Tetsuro Kitamura, Masatsugu Ikeuchi, Megumi Matsuda, Kazuhiko Yamamuro, Mai Sugimoto, Yasuyo Kobayashi, Yusuke Inagaki, Takashi Okada, Akira Kido

**Affiliations:** 1 Department of Rehabilitation Medicine, Nara Medical University, Kashihara, JPN; 2 Department of Neurosugery, Nara Medical University Hospital, Kashihara, JPN; 3 Psychiatric Center, Nara Medical University Hospital, Kashihara, JPN; 4 Center for Health Control, Nara Medical University Hospital, Kashihara, JPN; 5 Department of Psychiatry, Nara Medical University, Kashihara, JPN

**Keywords:** dementia care team, neurocognitive disorders, physical restraints, rehabilitation, training intensity

## Abstract

Background

In 2016, the Japanese health insurance system introduced a new dementia care incentive requiring clinical rounds by a dementia care team to promote dementia care in acute hospitals. Due to the nature of acute care, this round is conducted for all patients with neurocognitive disorders, including those with delirium and dementia. Both physical and psychological interventions are important, but the status of physical interventions for these patients is still unclear. This study investigated the current status by focusing on the intensity of rehabilitation training and the level of physical restraint as indicators of physical interventions.

Methods

Data from dementia care team rounds conducted between July 1, 2021, and October 7, 2024, were reviewed for relationships between clinicopathological characteristics, training programs for rehabilitation treatment, and physical restraint status.

Results

A total of 596 patients with neurocognitive disorders underwent 1,987 consultations. Patients with dementia, delirium, and a combination of the two were included. By disease groups, the frequency of consultations was higher than average for disuse, cardiovascular, and respiratory diseases, but lower for cerebrovascular, cancer, and musculoskeletal diseases. The training intensity was primarily level 1 (in-bed exercise) or level 5 (gait training) across all groups. A statistically significant relationship was observed between the disease group and the training intensity (p < 0.0001). The physical restraint (PR) level was significantly associated with the intensity of training in rehabilitation therapy (p < 0.0001).

Conclusions

The incidence of neurocognitive disorders varied according to the disease group. This intensity correlated with the number of patients subjected to physical restraint. To establish a care system, we should consider the characteristics of disease groups.

## Introduction

In Japan, considered a super-aged society, changes in the population structure have contributed to a rise in the number of elderly individuals with dementia, even within acute care hospitals [1,2]. To cover the costs of providing appropriate care for these elderly patients with dementia, the 2016 revision of the medical fee within the Japanese health insurance system introduced a new dementia care fee as an incentive to the basic hospitalization fee [3-5].

This additional payment can be calculated on a daily basis throughout the hospitalization period for elderly patients with dementia with level III or higher independence in daily living (excluding those with severe disorders of consciousness) [6]. To receive this additional payment, a dementia care team must be established and care conferences and ward rounds held at least once a week to improve the quality of dementia care [3,4]. It also stipulates that the team must be composed of full-time physicians with sufficient experience in the treatment of patients with dementia (either at least five years of experience in psychiatry, at least five years of experience in neurology, or completion of appropriate training), full-time nurses with at least five years of experience in the care of patients with dementia and who have completed relevant training in dementia care (at least 600 hours), and full-time social workers or full-time psychiatric social workers with experience in coordinating the discharge of patients with dementia [3,4]. Furthermore, depending on the patient’s condition, the inclusion of physiotherapists, occupational therapists, pharmacists, and dieticians is desirable [3,4].

Several groups have reported the effects of this overhaul, including both health and economic aspects [5,7,8]. Furthermore, the 2024 revision of medical fees identified the need to address delirium in dementia care while emphasizing the importance of strengthening initiatives to minimize physical restraint [9]. Physical restraints directly suppress physical activity, restricting the expressive movements of patients with dementia or delirium and hindering training as a treatment. Therefore, we consider it essential to investigate the condition of physical restraints alongside the state of physical intervention in these patients. Many groups have reported on the benefits of dementia care during acute treatment, even before this revision [10,11].

Dementia complicates acute-phase treatment. However, acute care hospitals are not primarily designed to provide specialized care for these patients, nor are they admitted with the primary objective of managing neurocognitive disorders [12,13]. Consequently, despite its clinical significance, the incidence and characteristics of neurocognitive disorders in acute care settings remain insufficiently studied. Since appropriate dementia care offers several advantages to executing acute medical treatment, we believe a physical intervention approach is critical to effective dementia care.

When treating neurocognitive disorders in the acute setting, it is important to adopt an approach that includes the management of patients with delirium. Delirium is a reversible disturbance of consciousness that can be caused by various factors, including medication and surgical trauma. Patients with dementia are at a high risk of developing delirium, and it is often difficult to distinguish between the two clinically [14]. Therefore, it is necessary to treat patients with delirium during the acute care of dementia.

As part of implementing the dementia care incentive, our hospital, like many acute care hospitals, began dementia care team rounds in April 2017, as required by regulations. The team included a psychiatrist, a certified nurse practitioner specializing in dementia care, and a psychiatric social worker. In addition, as a unique initiative for our hospital, physiatrists, as of April 2021, physical therapists, and occupational therapists were also included in the team. Before they joined the dementia care team, the rounds were conducted in the typical style of psychiatric care, primarily focusing on medical interviews that included questions about sleep patterns and adjustments to psychotropic medications instead of engaging patients in physical activities or rehabilitation treatments. Conversely, after the new rounds were implemented, assessments of patients' physical disabilities, rehabilitation task progress, and hindering factors were conducted, with clinical information systematically shared among the medical team.

Research has shown that both physical and mental stimulation throughout rehabilitation play a key role in maintaining cognitive function [15,16]. On the other hand, systematic dementia care team rounds in acute hospitals in Japan have started relatively recently, and the situation regarding physical interventions for these patients in acute hospitals is not clear.

This study focuses on the training intensity and the level of physical restraint as physical interventions. The purpose of this study is to clarify the status regarding these physical interventions for acute patients with neurocognitive disorders, and to clarify the characteristics of each disease.

## Materials and methods

In this study, the target population for dementia care team rounds is all inpatients for whom the charge nurse or certified dementia care nurse recognizes that neurocognitive disorders are a barrier to acute care, including both dementia and delirium patients. These patients are admitted for treatment of the underlying disease, not for treatment of the neurocognitive disorders. Rehabilitation for the underlying condition has begun prior to the round. In addition, the diagnosis of neurocognitive disorders has been confirmed by a psychiatrist who meets the prescribed requirements.

The level of independence in daily living for the elderly with dementia

Table 1 lists the English translations of the definitions for the different levels of independent living. This is an essential indicator in the certification of persons in need of long-term care under the Long-Term Care Insurance Law, which was enacted on October 26, 1993, as Notification No. 135 of the Director of the Health and Welfare Bureau for the Elderly, Ministry of Health, Labour and Welfare [6].

**Table 1:** Level of independence in the daily live of elderly people with dementia

Rank	Criteria
I	Patient has some form of dementia, but is largely independent in their daily life, both at home and socially.
II	Patient is able to live independently under the supervision of someone else, although there may be some symptoms, behaviors, or communication difficulties that interfere with daily life.
IIa	The symptoms of condition II are observed outside the home.
IIb	The symptoms of condition II are also be seen in the home.
III	Patient has symptoms or behaviors that interfere with their daily life, and they have difficulty communicating, and they require nursing care.
IIIa	The symptoms of condition III are seen mainly during the day.
IIIb	The symptoms of condition III are seen mainly at night.
IV	Patient frequently shows symptoms and behavior that interfere with daily life, as well as difficulties in communication, and always requires nursing care.
M	Patient shows significant psychiatric symptoms, problematic behavior, or serious physical illness and requires specialized medical care.

Patients included in the study

**Figure 1:**
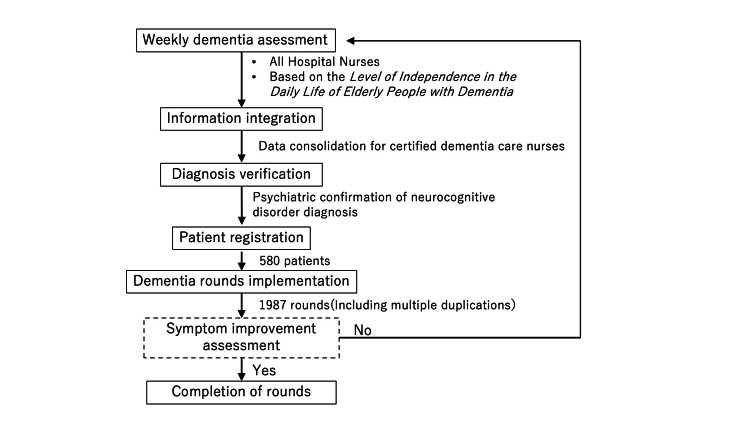
Flowchart of the case enrollment process.

Patient selection is based on the diagnosis of the certified dementia care nurses. The certified dementia care nurses supervise all nurses in the hospital and assess the cognitive function of all inpatients aged 65 years and older once a week. All patients with a rank III or higher neurocognitive disorder that is a barrier to acute treatment are selected for the round. The diagnosis is confirmed by a team psychiatrist (Figure 1). In addition, in our hospital, even if the patient is under 65 years old, adult patients diagnosed by nurses according to these criteria are also eligible. Patients under 20 years old, those with life-threatening events, and those due to be discharged before the next scheduled round were excluded from the study. A psychiatrist confirmed the diagnosis of the neurocognitive disorders in accordance with the Diagnostic and Statistical Manual of Mental Disorders, Fifth edition (DSM-5, American Psychiatric Association). The study utilized data from 1,987 patient rounds (580 patients) conducted by the dementia care team between July 1, 2021, and October 7, 2024. Information regarding age, sex, primary diagnosis, purpose of hospitalization, rehabilitation treatment program, and physical restraint status was extracted from medical records and analyzed. Written informed consent was obtained from patients. This study was approved by the Nara Medical University Ethics Committee (Research approval no. 3913).

Classification of disease groups

According to the Classification of Rehabilitation by Disease, diseases were grouped into six categories: musculoskeletal diseases, cancers, respiratory diseases, cardiovascular diseases, cerebrovascular diseases, and disuse syndromes [17]. The details of these are reported separately since cerebrovascular diseases include neuromuscular and spinal cord diseases. As disuse syndromes include several conditions, they were grouped into six categories to provide a more precise description. These include gastrointestinal diseases, kidney diseases, mental disorders, sepsis, infectious diseases, cholangitis, vasculitis, and systemic inflammatory diseases.

Rehabilitation treatment

Rehabilitation treatment information regarding ambulation training, muscle strengthening training, eating and swallowing function training, and higher brain function training (cognitive function training) was extracted from the medical records. The analysis of ambulation training was further divided into five levels in order of decreasing intensity: 1) therapeutic (in bed) exercise, 2) bed mobility (spine to sit) training, 3) transfer training, 4) standing training, and 5) gait training [18,19].

Physical restraints

We reviewed five types of restraints from medical records: 1) floor sensor mats, 2) pull-string alarms, 3) bedside rails, 4) hand restraint mittens, and 5) trunk restraints (belts). We classified 1 and 2 as mild restraints (I), and 3, 4, and 5 as severe restraints (II).

Statistical analysis

JMP Pro version 17.2.0 (SAS Institute Inc., Cary, NC, USA) was used for statistical analysis. The Kruskal-Wallis test was performed for age of the disease groups. Chi-square tests were performed as a nominal or ordinal scale for each disease group, ambulation training, and physical restraint content. Statistical significance was set at P < 0.05.

## Results

The attributes of patients categorized by disease group are presented in Table 2, while details on cerebrovascular disease and disuse syndrome are provided in Table 3. We found no statistical significance for age of disease groups (p=0.367).

**Table 2:** Patient characteristics

					Disease groups		
Variables	No. of patients	Musculoskeletal diseases	Cancer	Respiratory diseases	Cardiovascular diseases	Cerebrovascular diseases	Disuse syndormes
Gender							
Male	325	12	67	46	75	68	57
Female	255	26	28	21	35	89	56
Age (SD)	74.7 (15.3)	85.7 (13.5)	75.0 (6.7)	79.8 (13.2)	77.5 (22.0)	71.6 (13.8)	66.6 (23.7)

**Table 3:** Subgroup characteristics

Disease groups		No. of patients
Cerebrovascular diseases		157
	Cerebrovascular diseases	124
	Spinalcord diseases	18
	Neuromuscular diseases	15
Disuse syndrome		113
	Gastrointestinal diseases	34
	Kidney diseases	16
	Mental disorders	15
	Sepsis/Infectious diseases	15
	Cholangitis	13
	Vasculitis/Systemic inflammatory disease	6
	Others	14

**Figure 2:**
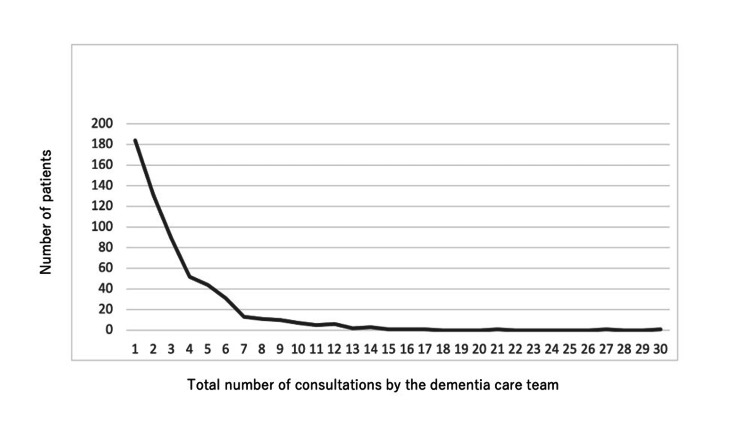
Number of patients and consultations by the dementia care team. A total of 1,987 rounds of consultations were conducted in 580 patients.

Figure 2 shows the relationship between the total number of consultations and the number of patients among the 580 patients who received consultations during the relevant period. A total of 1,987 rounds of consultation were conducted during the study period with 580 patients. Many of the patients (approximately 31%) received only one consultation, and the number of patients decreased as the total number of consultations increased (maximum 30). Multiple consecutive visits indicated that the patient’s neurocognitive disorders hindered acute treatment, as assessed by the nurse in charge or a certified nurse for dementia. Conversely, a few rounds indicated that the patient’s condition had improved to the point where symptoms were no longer hindered.

Total number of consultations by disease group

**Figure 3:**
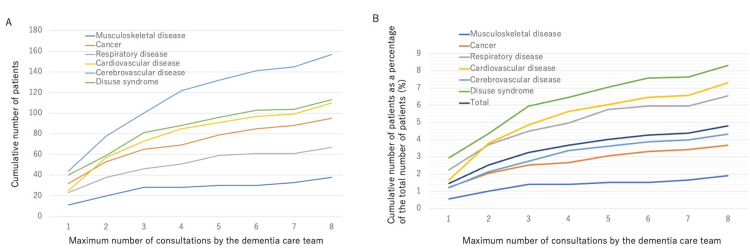
Cumulative number of patients and total number of consultations. (A) Cumulative number of patients and consultations by disease group. (B) Cumulative number of patients as a percentage of patients prescribed rehabilitation by diseases.

The total number of consultations per disease group is detailed in Figure 3A. The number of patients in each disease group gradually increased with the maximum number of consultations. The total number of patients was as follows: cerebrovascular, disuse, cardiocerebrovascular, cancer, respiratory, and musculoskeletal diseases. Figure 3B shows the percentage of this value divided by the total number of rehabilitation prescriptions per disease group during the study period. The percentage of all patients who received this consultation divided by the total number of rehabilitation prescriptions is also shown as a control. Interestingly, the adjusted values showed that the number of patients with disuse and cardiovascular and respiratory disorders exceeded the control values, whereas the number of patients with cerebrovascular, cancer, and musculoskeletal disorders decreased in that order.

Relationship between the training intensity and disease group

**Figure 4:**
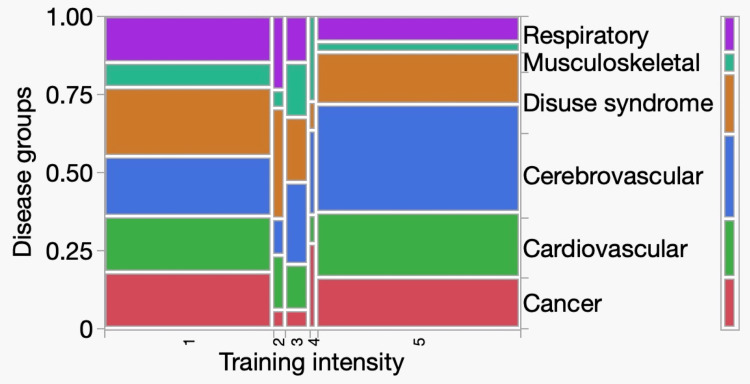
Analysis of training intensity and disease groups contingency table

Table 4 shows the relationship between training intensity and disease group. Interestingly, levels 1 and 5 were the most common in all disease groups. Level 5 was the most common cerebrovascular disease, and level 1 was the most common respiratory disease. This suggests that in situations where neurocognitive disorders are a barrier to acute treatment, many patients are only able to train in bed or are already able to walk, and few are undergoing training to acquire sitting and transfer skills. In addition, there was a statistically significant relationship between the disease group and the training intensity (p<0.0001). Figure 4 shows the mosaic plot.

**Table 4:** Training intensity and disease groups

Disease groups	Training intensity (no. patients, %)				
	Level 1	Level 2	Level 3	Level 4	Level 5
Musculoskeletal	18 (47.4)	1 (2.6)	6 (15.8)	3 (7.9)	10 (26.3)
Cancer	43 (45.3)	1 (1.1)	2 (2.1)	3 (3.2)	46 (48.4)
Respiratory	35 (52.2)	4 (6.0)	5 (7.5)	0 (0)	23 (34.3)
Cardiovascular	42 (38.2)	3 (2.7)	5 (4.5)	1 (0.9)	59 (53.6)
Cerebrovascular	45 (28.7)	2 (1.3)	9 (5.7)	3 (1.9)	98 (62.4)
Disuse syndorme	52 (46.0)	6 (5.3)	7(6.2)	1 (0.9)	47 (41.6)

Physical restraint and training intensity

**Figure 5:**
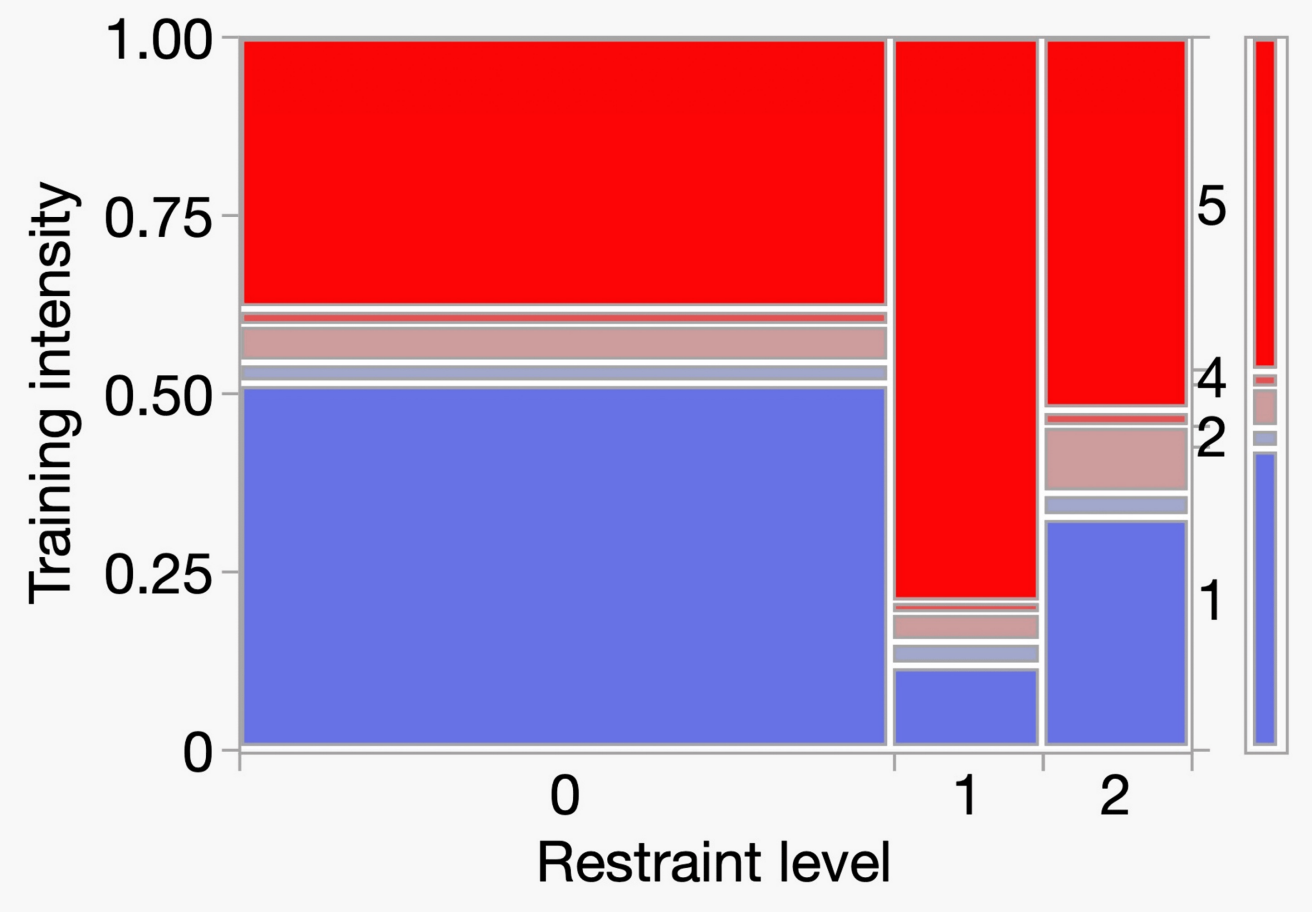
Analysis of restraint level and training intensity contingency tables

Next, we investigated the relationship between physical restraint and training intensity. Physical restraint was significantly associated with training intensity. We found that more patients who received physical restraint received high-intensity training than those who received low-intensity training (Table 5) (p<0.0001). Figure 5 shows the mosaic plot.

**Table 5:** Training intensity and physical restraints

Variables	Training intensity (no. patients, %)				
	Level 1	Level 2	Level 3	Level 4	Level 5
No restraints	209 (51.5)	11 (2.71)	22 (5.42)	9 (2.22)	155 (38.18)
Restraints (I)	11 (11.83)	3 (3.2)	4 (4.3)	1 (1.0)	74 (79.6)
Restraints (II)	30 (32.6)	3 (3.3)	9 (9.8)	2 (2.2)	48 (52.2)
(I) Mild restraints. (II) Severe restraints.					

## Discussion

There is no doubt that neurocognitive disorders are a barrier to acute care. Adequate care is expected to contribute to patients’ quality of life by ensuring that acute treatment proceeds immediately. Dementia care rounds initiated in response to the revision of medical fees are aimed at helping patients overcome various problems to ensure quality of care. To provide better care, this study focused on using information related to the implementation of rehabilitation treatments. By studying the characteristics of the rehabilitation treatments in patients with neurocognitive disorders and clarifying the issues involved, this study provides insights useful for the development of new approaches aimed at improving the care environment of these patients.

In the present study, the number of patients who underwent the rounds and the total number of consultations varied significantly depending on the disease group. This suggests that different disease characteristics of the acute phase are related to the onset of neurocognitive disorders.

In addition, there were significant differences in the intensity of ambulation training during the rehabilitation treatment for each disease group. In the cerebrovascular disease, cardiovascular disease, and cancer groups, many participants engaged in high-intensity training, whereas in the other disease groups, many participated in low-intensity training. Many patients received level 1 or 5 training, suggesting that in situations where neurocognitive disorders are a barrier to acute treatment, many patients could only receive training in bed or were already able to walk. Few received training in learning to sit or transfer. In general, regardless of disease group, training is expected to be conducted according to the patient’s condition and age, without bias toward a specific intensity. Therefore, the observed deviation in training intensity in this study may be specific to patients with neurocognitive disorders.

Delirium is critical when analyzing neurocognitive disorders in acute patients. Delirium often occurs in patients with dementia, and the various symptoms of early delirium are similar to the behavioral and psychological symptoms of dementia (BPSD), making it difficult to distinguish between the two. Therefore, these conditions are typically treated without differentiation. The most crucial difference between dementia and delirium is that the onset of delirium is sudden, with the changes occurring over several days [14]. In contrast to the onset of BPSD, the causes of delirium are often clear, and the temporal relationship between changes in physical status and the effects of medications is often easy to understand [14]. However, it is believed that the acute phase of treatment is where changes in physical condition and the effects of medication occur, and it is difficult to accurately distinguish between them; therefore, in this study, all patients who presented with neurocognitive disorders were included.

The results regarding physical restraint were consistent with the general assumptions. The patients who were more active were more likely to be physically restrained than those who were less active. Logically, it is easy to understand that those who can move more are at higher risk and that physical restraint is necessary when there are neurocognitive disorders. However, the fact that those with greater functional capacity are more likely to be physically restrained suggests that those with greater capacity are being made to suffer, which is a profound humanitarian issue. Physical restraints directly suppress physical activity. In patients with dementia or delirium, they inhibit both the expression of intent through movement and physical training as a therapeutic intervention for these conditions. Therefore, it is crucial to assess the state of physical restraints in these patients.

Acute hospitals are generally not designed to provide the best care for neurocognitive disorders [12], and patients are not admitted for the treatment of the neurocognitive disorder itself [13]. In other words, neurocognitive disorders are not a priority for care or treatment. In addition, it has been reported that staff training and knowledge of care in acute hospitals is inadequate [12], which may result in unmet needs and increased BPSD [20]. From this perspective, this study provides baseline data from a rehabilitation medicine perspective to answer the question of what constitutes the best care.

This study had several limitations. First, it constitutes a report from a single institution. Second, this was a cross-sectional study; therefore, it is impossible to discuss causal relationships. Third, the disease classification was the classification of rehabilitation by disease category used by the Ministry of Health, Labour, and Welfare. This study used this classification from the perspective of examining the characteristics of rehabilitation treatment, which made it easy to compare the total number of patients in the hospital. In addition, to supplement the classification, we included details of cerebrovascular rehabilitation for cerebrovascular, spinal cord, and neuromuscular diseases, as well as more information on various diseases included in the disuse syndrome category. The fourth limitation is the number of cases. For items with expected frequencies of less than five in the test, there is a possibility of a problem with the chi-square test. However, the main items satisfied the accuracy requirements. Given the results of this study, we were most interested in the characteristics of patients with cardiovascular disease. Open-heart surgery has been reported to be a high-risk factor for delirium [21,22]. In this study, cardiovascular disease was the second most common condition among all patients (Figure 3B). In the future, we would like to perform a subgroup analysis of the presence or absence of surgery and surgical procedures. Although disuse syndrome was the most common, we would be interested in classifying these diseases. Among the other categories, cholangitis tended to be associated with a high incidence of neurocognitive disorders (data not shown); a statistical analysis of these subgroups is currently underway.

## Conclusions

Acute hospitals are not specifically designed to provide optimal care for patients with neurocognitive disorders, nor are these patients admitted primarily for their management. The incidence of neurocognitive disorders in acute care settings has not been sufficiently analyzed. The assessment framework presented in this study represents an innovative approach in which nurses, who are closely involved in patients’ daily lives, conduct regular weekly evaluations, screen patients, and refer them to certified nurses without waiting for a psychiatrist’s consultation.

Neurocognitive disorders pose a challenge in acute care. The incidence of neurocognitive disorders and the intensity of rehabilitation training in acute hospitals vary by disease group, and training intensity correlates with the level of physical restraint. Various characteristics of acute-phase diseases are associated with the onset of neurocognitive disorders. Dementia rounds should take into account the disease-specific physical characteristics of patients. Identifying these characteristics through dementia rounds is essential for selecting the appropriate rehabilitation intensity and reducing the use of physical restraints.

## References

[1] Ishihara M, Matsunaga S, Islam R, Shibata O, Chung UI (2024). A policy overview of Japan's progress on dementia care in a super-aged society and future challenges. Glob Health Med.

[2] Nakanishi M, Okumura Y, Ogawa A (2018). Physical restraint to patients with dementia in acute physical care settings: effect of the financial incentive to acute care hospitals. Int Psychogeriatr.

[3] (2024). Overview of FY2016 medical fee revisions. https://www.mhlw.go.jp/file/06-Seisakujouhou-12400000-Hokenkyoku/0000115977.pdf.

[4] (2024). Health and Medical Services. https://www.mhlw.go.jp/english/wp/wp-hw10/dl/02e.pdf.

[5] Morioka N, Moriwaki M, Tomio J, Kashiwagi M, Fushimi K, Ogata Y (2020). Structure and process of dementia care and patient outcomes after hip surgery in elderly people with dementia: a retrospective observational study in Japan. Int J Nurs Stud.

[6] (2024). Level of independence in daily life for elderly people with dementia. https://www.mhlw.go.jp/file/06-Seisakujouhou-12300000-Roukenkyoku/0000077382.pdf.

[7] Morita K, Fukahori H, Ogawara H (2021). Outcomes of a financial incentive scheme for dementia care by dementia specialist teams in acute-care hospitals: a difference-in-differences analysis of a nationwide retrospective cohort study in Japan. Int J Geriatr Psychiatry.

[8] Kawabata J, Fukuda H (2023). Effects of a financial incentive scheme for dementia care on medical and long-term care expenditures: a propensity score-matched analysis using LIFE study data. PLoS One.

[9] (2024). Ministry of Health, Labour and Welfare. In Overview of FY2020 medical fee revisions priority areas II (dementia, mental health care, medical care for patients with incurable diseases).. https://www.mhlw.go.jp/content/12400000/001238907.pdf..

[10] Timmons S, O'Shea E, O'Neill D (2016). Acute hospital dementia care: results from a national audit. BMC Geriatr.

[11] Guijarro R, San Román CM, Gómez-Huelgas R (2010). Impact of dementia on hospitalization. Neuroepidemiology.

[12] Borbasi S, Jones J, Lockwood C, Emden C (2006). Health professionals' perspectives of providing care to people with dementia in the acute setting: toward better practice. Geriatr Nurs.

[13] Park M, Delaney C, Maas M, Reed D (2004). Using a Nursing Minimum Data Set with older patients with dementia in an acute care setting. J Adv Nurs.

[14] Feast AR, White N, Lord K, Kupeli N, Vickerstaff V, Sampson EL (2018). Pain and delirium in people with dementia in the acute general hospital setting. Age Ageing.

[15] Fleiner T, Dauth H, Gersie M, Zijlstra W, Haussermann P (2017). Structured physical exercise improves neuropsychiatric symptoms in acute dementia care: a hospital-based RCT. Alzheimers Res Ther.

[16] Sáez de Asteasu ML, Martínez-Velilla N, Zambom-Ferraresi F, Casas-Herrero Á, Cadore EL, Galbete A, Izquierdo M (2019). Assessing the impact of physical exercise on cognitive function in older medical patients during acute hospitalization: secondary analysis of a randomized trial. PLoS Med.

[17] (2024). Overview of the FY2020 medical fee revision, Individual revision III. (Pediatric/perinatal, cancer/disease/incurable disease measures, rehabilitation). https://www.nhpta.net/cms/wp-content/uploads/2022/03/R3.3.4-riha.pdf.

[18] Kim RY, Murphy TE, Doyle M (2019). Factors associated with discharge home among medical ICU patients in an early mobilization program. Crit Care Explor.

[19] Ishida Y, Shigematsu H, Tsukamoto S (2022). Case series of an impairment driven early ambulation program in cancer patients with cervical spine metastases after palliative spine surgery. J Cancer Rehab.

[20] (2024). Counting the cost: Caring for people with dementia on hospital wards. https://www.alzheimers.org.uk/sites/default/files/2018-05/Counting_the_cost_report.pdf.

[21] Petersson NB, Hansen MH, Hjelmborg JV (2024). Incidence and assessment of delirium following open cardiac surgery: a systematic review and meta-analysis. Eur J Cardiovasc Nurs.

[22] Greaves D, Psaltis PJ, Ross TJ, Davis D, Smith AE, Boord MS, Keage HA (2019). Cognitive outcomes following coronary artery bypass grafting: a systematic review and meta-analysis of 91,829 patients. Int J Cardiol.

